# Supplementary material for: Lacticaseibacillus rhamnosus Probio-M9 extends the lifespan of Caenorhabditis elegans
Source: Commun Biol. 2022 Oct 27;5:1139. doi: 10.1038/s42003-022-04031-2 (PMC9613993; doi:10.1038/s42003-022-04031-2)
Supplement: Supplementary file 2 — Supplementary Information [file 42003_2022_4031_MOESM2_ESM.pdf]

Supplementary Information

Lacticaseibacillus rhamnosus Probio-M9 Extends the Lifespan of *Caenorhabditis elegans*

Juntao Zhang[1,2,3], Yanmei Zhao[4], Zhihong Sun[1,2,3], Tiansong Sun[1,2,3*]

[1]Inner Mongolia Key Laboratory of Dairy Biotechnology and Engineering, Inner Mongolia Agricultural University, Hohhot, Inner Mongolia, China

[2]Key Laboratory of Dairy Products Processing, Ministry of Agriculture and Rural Affairs, Inner Mongolia Agricultural University, Hohhot, Inner Mongolia, China

[3]Key Laboratory of Dairy Biotechnology and Engineering, Ministry of Education, Inner Mongolia Agricultural University, Hohhot, Inner Mongolia, China

[4]Key Laboratory of RNA Biology, CAS Center for Excellence in Biomacromolecules, Institute of Biophysics, Chinese Academy of Sciences, Beijing, China

*To whom correspondence should be addressed:
E-mail: sts9940@sina.com

Running Title: Lifespan Extension by Probio-M9 in *Caenorhabditis elegans*

Key Words: Insulin/IGF-1 signaling pathway, p38 MAPK signaling pathway, Stress response, Mitochondrial unfolded protein response, *Lacticaseibacillus rhamnosus*, *Caenorhabditis elegans*

This file includes:

Supplementary Figures 1-4

Supplementary Tables 1-8

Supplementary Figures

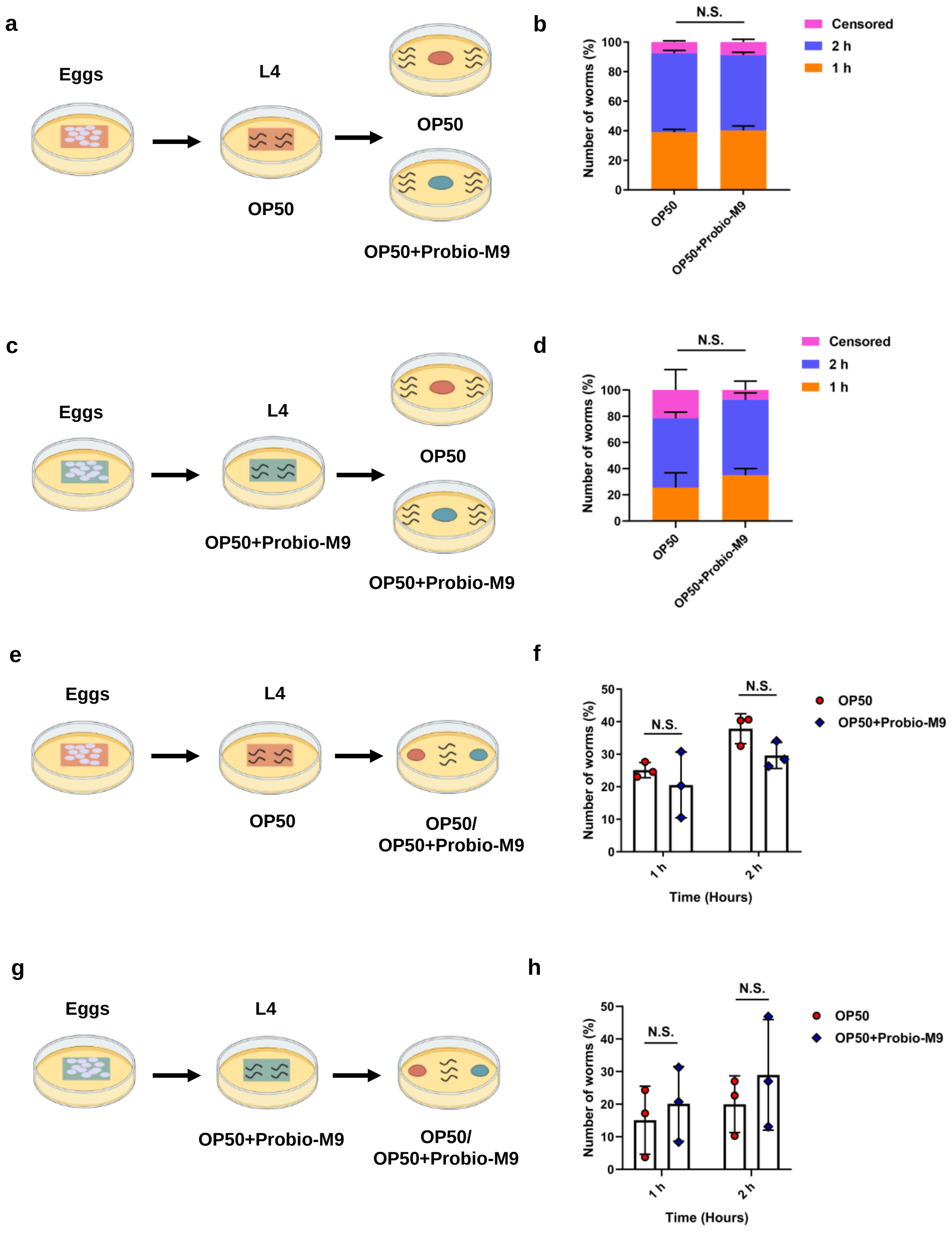

Supplementary Figure 1. Bacterial choice assay of *C. elegans*. **a**, **c**, **e**, **g** indicated the schematics illustrate the method and bacteria used in each assay. The “Number of worms” which is the average percentage of worms (±SEM) inside the specified bacterial lawn was shown in **b**, **d**, **f**, **h**. Censored, the number of outside the specified bacterial lawn. The detail procures see the “methods” ($p > 0.05$, Chi-squared test). In **b** and **d**, N = 100 worms. In **f** and **h**, N = 3 biologically experiments.

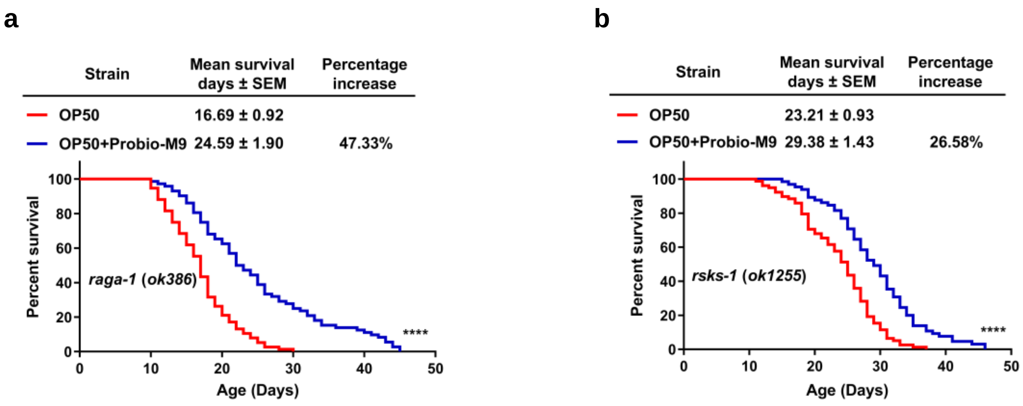

Supplementary Figure 2. Probio-M9-induced lifespan extension was independent of TOR. a, **b** Survival curves of mutants of TOR signaling pathway, *raga-1* (*ok386*) (**a**) and *rsks-1* (*ok1255*) (**b**) fed with Probio-M9 (N = 90 worms, $p < 0.0001$, Log rank test).

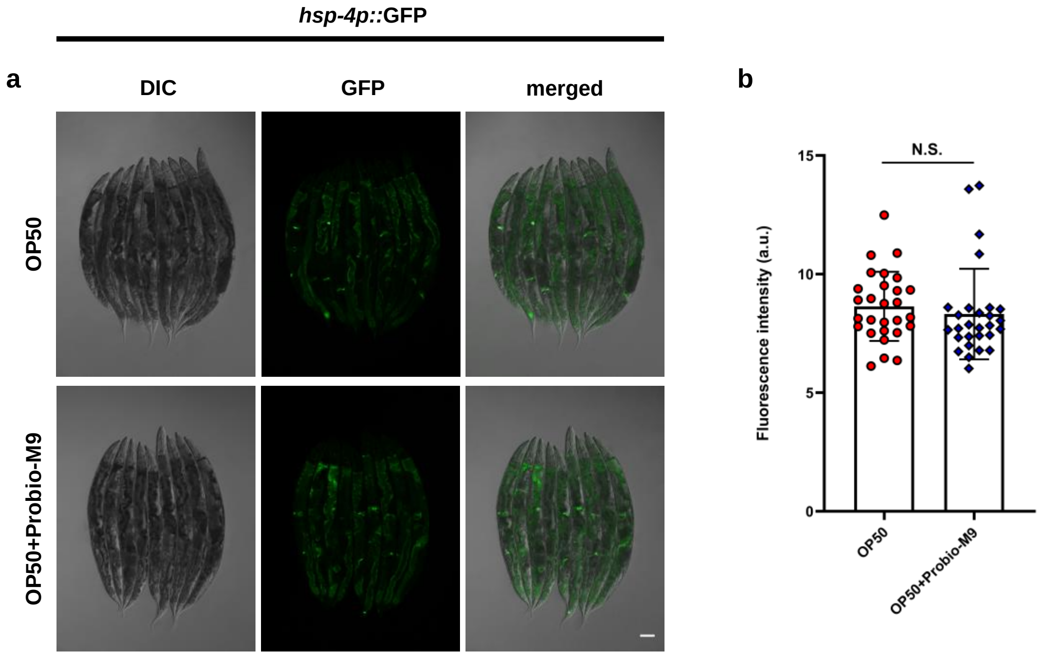

Supplementary Figure 3. Probio-M9 has no effect on the induction of UPR^{ER}. a The stress reporter *hsp-4p::*GFP was used to measure UPR^{ER}. Worms were treated with 50 ng/mL tunicamycin or DMSO and rotated in a rotator at 20°C for 4 h induces *hsp-4p::*GFP reporter. Scale bar, 20 μm. **b** Fluorescence intensity of GFP was quantified using ImageJ software. Probio-M9 has no influence on UPR^{ER} responding stress induction (N = 28 worms, values are presented as the mean ± SEM; $p > 0.05$, Student's t test).

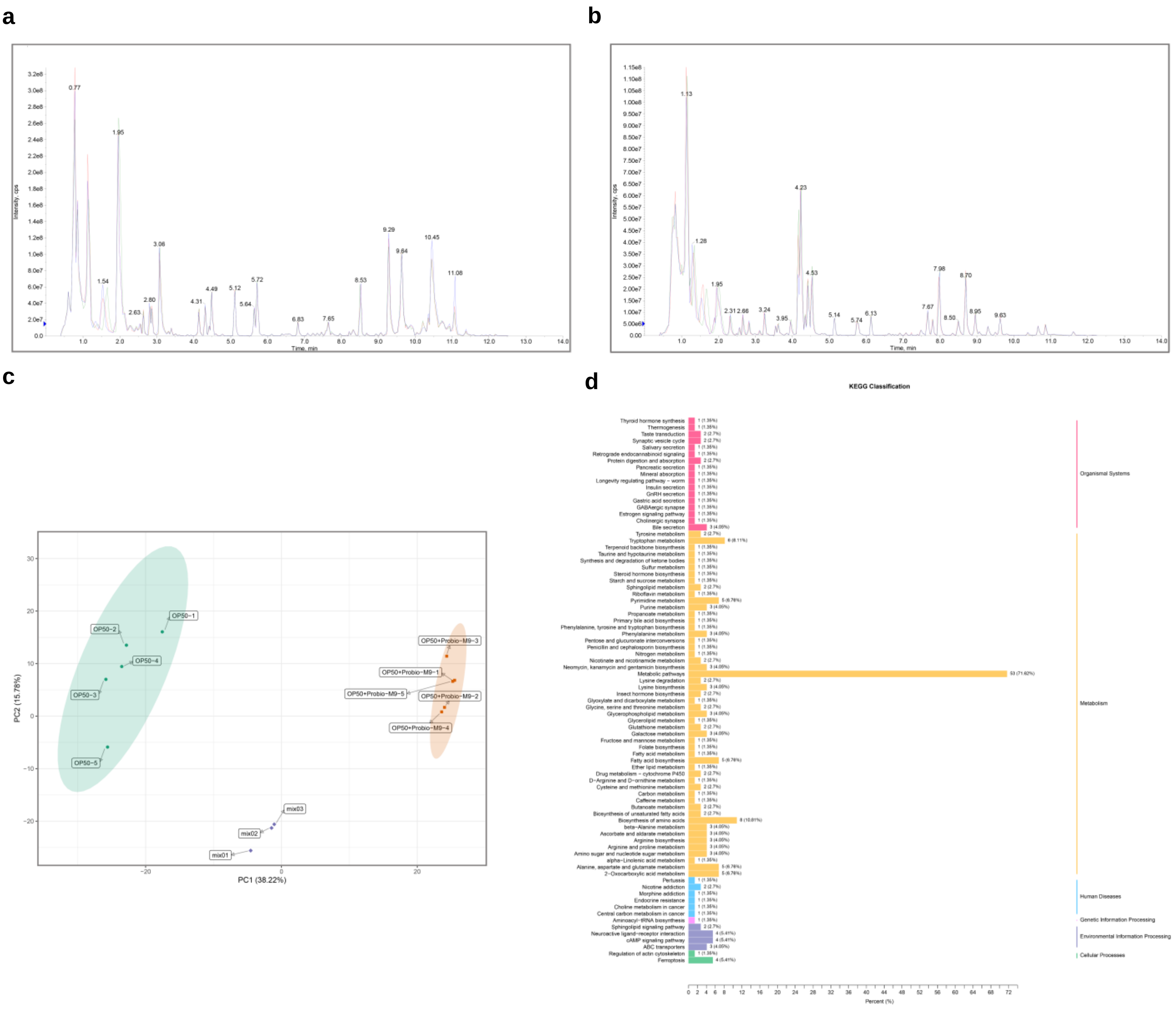

Supplementary Figure 4. Quality control of metabolomics and classification chart of KEGG pathways. a, **b** The superposition of total ions chromatography (TIC) graphs. **a** presents positive TIC and **b** presents negative TIC. These showed high level of superposition when comparing the retention time and intensity across of each chromatographic peak, and high instrument stability. **c** Sore plots of principal components

analysis for quality control (QC) and samples. PCA scores showed there was an obvious separation between OP50 and OP50+Probio-M9, and QC bacteria (mix01, mix02 and mix03) were able to gather together. The repeatability of the experimental conditions and data obtained were stable and reliable. **d** Classification graph of KEGG pathways annotated with significantly differential metabolites. The abscissa indicates the proportion of total number of metabolites were annotated in this pathway. The ordinate indicates the names of the KEGG metabolic pathways were identified.

Supplementary Table 1. Lifespan analysis related to Figure 1.

C. elegans strain	Repeats	Total N (censor N)	Strain	Mean ± SEM (days)	Lifespan Extension	*p*-value
N2	1	90 (3)	OP50	14.85 ± 0.60	control	
		90 (4)	OP50+Probio-M9	19.63 ± 1.05	32.19%	< 0.0001
N2	2	90 (18)	OP50	15.65 ± 1.10	control	
		90 (20)	OP50+Probio-M9	20.93 ± 1.28	33.74%	< 0.0001
N2	3	90 (6)	OP50	15.69 ± 1.01	control	
		90 (7)	OP50+Probio-M9	19.50 ± 1.09	24.28%	< 0.0001
N2	1	90 (13)	OP50	12.85 ± 0.67	control	
		90 (17)	OP50+0.04 Probio-M9	14.33 ± 0.82	11.52%	0.0036
		90 (16)	OP50+0.4 Probio-M9	14.38 ± 0.84	11.91%	0.0068
		90 (13)	OP50+4 Probio-M9	16.57 ± 0.94	28.95%	< 0.0001
		90 (22)	OP50+40 Probio-M9	14.53 ± 0.74	13.07%	0.0063
N2	2	90 (4)	OP50	13.04 ± 0.68	control	
		90 (11)	OP50+0.04 Probio-M9	15.02 ± 0.94	15.18%	0.0085
		90 (9)	OP50+0.4 Probio-M9	14.28 ± 0.82	9.51%	0.0464
		90 (8)	OP50+4 Probio-M9	17.33 ± 1.08	32.90%	< 0.0001
		90 (12)	OP50+40 Probio-M9	14.88 ± 0.69	14.11%	0.0092
N2	3	90 (5)	OP50	13.29 ± 0.69		
		90 (9)	OP50+0.04 Probio-M9	13.65 ± 0.70	2.71%	0.7107
		90 (12)	OP50+0.4 Probio-M9	13.78 ± 0.69	3.69%	0.5658
		90 (6)	OP50+4 Probio-M9	16.57 ± 0.94	24.68%	< 0.0001
		90 (27)	OP50+40 Probio-M9	13.71 ± 0.65	3.16%	0.6215

Total N, the number of total animals; censor N, the number of censored animals; Lifespan extension, the percentage of lifespan extension in wild-type N2 worms on OP50+Probio-M9 compared to those on OP50; *p* values, OP50+Probio-M9 versus OP50 by log rank test.

Supplementary Table 2. Lifespan analysis related to Figure 2.

C. elegans strain	Repeats	Total N (censor N)	Strain	Mean ± SEM (days)	Lifespan Extension	*p*-value
eat-2 (*ad1116*)	1	90 (26)	OP50	20.33 ± 1.30	control	
		90 (25)	OP50+Probio-M9	25.67 ± 1.43	26.27%	< 0.0001
eat-2 (*ad1116*)	2	90 (24)	OP50	19.18 ± 1.24	control	
		90 (27)	OP50+Probio-M9	22.85 ± 1.52	19.13%	< 0.0001
eat-2 (*ad1116*)	3	90 (12)	OP50	19.70 ± 1.33	control	
		90 (20)	OP50+Probio-M9	24.43 ± 1.54	24.01%	< 0.0001
aak-2 (*ok524*)	1	90 (8)	OP50	14.64 ± 1.02	control	
		90 (11)	OP50+Probio-M9	18.03 ± 1.72	23.16%	< 0.0001
aak-2 (*ok524*)	2	90 (11)	OP50	14.03 ± 0.89	control	
		90 (6)	OP50+Probio-M9	18.58 ± 1.25	32.43%	< 0.0001
aak-2 (*ok524*)	3	90 (29)	OP50	14.23 ± 0.93	control	
		90 (22)	OP50+Probio-M9	18.40 ± 1.41	29.30%	< 0.0001

Total N, the number of total animals; censor N, the number of censored animals; Lifespan extension, the percentage of lifespan extension in different *C. elegans* mutants on OP50+Probio-M9 compared to those on OP50; *p* values, OP50+Probio-M9 versus OP50 by log rank test.

Supplementary Table 3. Lifespan analysis related to Figure 3.

C. elegans strain	Repeats	Total N (censor N)	Strain	Mean ± SEM (days)	Lifespan Extension	*p*-value
N2 (Switch to OP50 on L4)	1	90 (6)	OP50	14.43 ± 0.70	control	
		90 (9)	OP50+Probio-M9	15.69 ± 0.77	8.73%	0.0475
N2 (Switch to OP50 on L4)	2	90 (4)	OP50	13.92 ± 0.69	control	
		90 (5)	OP50+Probio-M9	15.29 ± 0.85	9.84%	0.0348
N2 (Switch to OP50 on L4)	3	90 (6)	OP50	14.43 ± 0.70	control	
		90 (6)	OP50+Probio-M9	15.46 ± 0.93	7.14%	0.0510
N2 (Switch to OP50 on Day 2 after L4)	1	90 (2)	OP50	12.38 ± 0.88	control	
		90 (3)	OP50+Probio-M9	14.14 ± 0.71	14.21%	0.0079
N2 (Switch to OP50 on Day 2 after L4)	2	90 (4)	OP50	14.07 ± 0.80	control	
		90 (5)	OP50+Probio-M9	15.30 ± 1.07	8.74	0.0435
N2 (Switch to OP50 on Day 2 after L4)	3	90 (10)	OP50	12.69 ± 0.81	control	
		90 (10)	OP50+Probio-M9	14.52 ± 0.82	14.42%	0.0066
N2 (Switch to OP50 on Day 4 after L4)	1	90 (2)	OP50	11.56 ± 0.69	control	
		90 (11)	OP50+Probio-M9	13.15 ± 0.93	13.75%	0.0092
N2 (Switch to OP50 on Day 4 after L4)	2	90 (0)	OP50	12.06 ± 0.78	control	
		90 (4)	OP50+Probio-M9	14.10 ± 1.10	16.92%	0.0043
N2 (Switch to OP50 on Day 4 after L4)	3	89 (1)	OP50	11.43 ± 0.79	control	
		90 (3)	OP50+Probio-M9	13.68 ± 0.83	19.68%	0.0008
N2	1	90 (12)	OP50	13.27 ± 0.84	control	
		90 (19)	OP50+Heat-inactived Probio-M9	13.96 ± 0.97	5.20%	0.2625
N2	2	90 (5)	OP50	12.38 ± 0.64	control	
		90 (6)	OP50+Heat-inactived Probio-M9	13.18 ± 0.97	6.46%	0.3341
N2	3	90 (4)	OP50	13.82 ± 0.94	control	
		90 (5)	OP50+Heat-inactived Probio-M9	14.21 ± 1.00	2.82%	0.7885

Total N, the number of total animals; censor N, the number of censored animals; Lifespan extension, the percentage of lifespan extension in wild-type N2 worms on OP50+Probio-M9 or OP50+Heat-inactived Probio-M9 compared to those on OP50; *p* values, OP50+Probio-M9 or OP50+Heat-

inactived Probio-M9 versus OP50 by log rank test.

Supplementary Table 4. Lifespan analysis related to Figure 4.

C. elegans strain	Repeats	Total N (censor N)	Strain	Mean ± SEM (days)	Lifespan Extension	*p*-value
nsy-1 (*ag3*)	1	90 (30)	OP50	11.75 ± 1.80	control	
		90 (15)	OP50+Probio-M9	11.07 ± 0.92	-5.79%	0.4728
nsy-1 (*ag3*)	2	90 (22)	OP50	11.41 ± 0.89	control	
		90 (17)	OP50+Probio-M9	11.75 ± 1.49	2.98%	0.5564
nsy-1 (*ag3*)	3	90 (30)	OP50	11.58 ± 1.34	control	
		90 (30)	OP50+Probio-M9	11.41 ± 1.20	-1.50%	0.8131
sek-1 (*km4*)	1	90 (24)	OP50	12.00 ± 0.50	control	
		90 (16)	OP50+Probio-M9	13.19 ± 0.90	9.92%	0.1083
sek-1 (*km4*)	2	90 (18)	OP50	11.77 ± 0.50	control	
		90 (24)	OP50+Probio-M9	13.14 ± 0.80	11.64%	0.1299
sek-1 (*km4*)	3	90 (22)	OP50	11.56 ± 0.56	control	
		90 (13)	OP50+Probio-M9	13.10 ± 0.69	13.32%	0.0526
pmk-1 (*km25*)	1	90 (12)	OP50	10.83 ± 0.85	control	
		90 (20)	OP50+Probio-M9	11.35 ± 0.71	4.80%	0.5966
pmk-1 (*km25*)	2	90 (13)	OP50	11.00 ± 0.64	control	
		90 (23)	OP50+Probio-M9	11.31 ± 0.86	2.82%	0.6524
pmk-1 (*km25*)	3	90 (20)	OP50	10.40 ± 0.65	control	
		90 (10)	OP50+Probio-M9	11.68 ± 0.78	12.31%	0.2999
tir-1 (*tm3036*)	1	90 (19)	OP50	16.23 ± 1.27	control	
		90 (9)	OP50+Probio-M9	16.36 ± 0.74	0.80%	0.7483
tir-1 (*tm3036*)	2	90 (14)	OP50	15.63 ± 1.14	control	
		90 (15)	OP50+Probio-M9	15.83 ± 1.20	1.28%	0.7953
tir-1 (*tm3036*)	3	90 (18)	OP50	15.49 ± 0.94	control	
		90 (18)	OP50+Probio-M9	16.10 ± 0.97	3.94%	0.2699
tir-1 (*ok1052*)	1	90 (9)	OP50	17.26 ± 0.88	control	
		90 (9)	OP50+Probio-M9	16.55 ± 0.77	-4.11%	0.3606
tir-1 (*ok1052*)	2	90 (4)	OP50	18.42 ± 0.62	control	
		90 (11)	OP50+Probio-M9	18.61 ± 0.86	1.03%	0.5906

Supplementary Table 4 (continue). Lifespan analysis related to Figure 4.

C. elegans strain	Repeats	Total N (censor N)	Strain	Mean ± SEM (days)	Lifespan Extension	*p*-value
tir-1 (*ok1052*)	3	90 (21)	OP50	17.86 ± 0.70	control	
		90 (32)	OP50+Probio-M9	17.75 ± 0.82	-0.62%	0.9717
atf-7 (*gk715*)	1	90 (22)	OP50	10.28 ± 0.95	control	
		90 (19)	OP50+Probio-M9	10.63 ± 1.29	3.40%	0.6235
atf-7 (*gk715*)	2	90 (21)	OP50	11.38 ± 0.83	control	
		90 (22)	OP50+Probio-M9	11.05 ± 0.87	-2.90%	0.9032
atf-7 (*gk715*)	3	90 (12)	OP50	11.24 ± 0.88	control	
		90 (17)	OP50+Probio-M9	11.66 ± 0.94	3.74%	0.3499

Total N, the number of total animals; censor N, the number of censored animals; Lifespan extension, the percentage of lifespan extension in different *C. elegans* mutants on OP50+Probio-M9 compared to those on OP50; *p* values, OP50+Probio-M9 versus OP50 by log rank test.

Supplementary Table 5. Lifespan analysis related to Figure 5.

C. elegans strain	Repeats	Total N (censor N)	Strain	Mean ± SEM (days)	Lifespan Extension	*p*-value
daf-2 (*e1370*)	1	90 (5)	OP50	47.21 ± 1.73	control	
		90 (9)	OP50+Probio-M9	47.15 ± 1.29	-0.13%	0.3761
daf-2 (*e1370*)	2	90 (10)	OP50	50.27 ± 1.60	control	
		90 (0)	OP50+Probio-M9	48.83 ± 1.38	-2.86%	0.2502
daf-2 (*e1370*)	3	90 (5)	OP50	49.39 ± 1.61	control	
		90 (3)	OP50+Probio-M9	51.16 ± 1.59	3.58%	0.9451
age-1 (*hx546*)	1	90 (6)	OP50	21.04 ± 1.38	control	
		90 (8)	OP50+Probio-M9	25.17 ± 1.00	19.63%	< 0.0001
age-1 (*hx546*)	2	90 (5)	OP50	22.18 ± 1.70	control	
		90 (7)	OP50+Probio-M9	26.65 ± 1.89	20.15%	< 0.0001
age-1 (*hx546*)	3	90 (16)	OP50	21.73 ± 1.46	control	
		90 (17)	OP50+Probio-M9	26.91 ± 1.48	23.84%	< 0.0001
daf-16 (*mgDf50*)	1	90 (12)	OP50	11.67 ± 0.41	control	
		90 (15)	OP50+Probio-M9	15.97 ± 0.54	36.85%	< 0.0001
daf-16 (*mgDf50*)	2	90 (8)	OP50	11.31 ± 0.53	control	
		90 (19)	OP50+Probio-M9	15.08 ± 0.48	33.33%	< 0.0001
daf-16 (*mgDf50*)	3	90 (16)	OP50	11.49 ± 0.47	control	
		90 (4)	OP50+Probio-M9	15.26 ± 0.51	32.81%	< 0.0001
skn-1 (*mg570*)	1	90 (19)	OP50	13.79 ± 0.96	control	
		90 (16)	OP50+Probio-M9	14.36 ± 1.29	4.13%	0.7326
skn-1 (*mg570*)	2	90 (20)	OP50	13.30 ± 0.94	control	
		90 (14)	OP50+Probio-M9	13.77 ± 0.93	3.53%	0.2323
skn-1 (*mg570*)	3	90 (33)	OP50	13.15 ± 0.90	control	
		90 (20)	OP50+Probio-M9	14.31 ± 1.02	8.82%	0.9595
hsf-1 (*sy441*)	1	90 (19)	OP50	11.98 ± 0.35	control	
		90 (22)	OP50+Probio-M9	11.39 ± 0.32	-4.92%	0.3438
hsf-1 (*sy441*)	2	90 (17)	OP50	12.05 ± 0.31	control	
		90 (14)	OP50+Probio-M9	12.56 ± 0.43	4.23%	0.0804

Supplementary Table 5 (continue). Lifespan analysis related to Figure 5.

C. elegans strain	Repeats	Total N (censor N)	Strain	Mean ± SEM (days)	Lifespan Extension	*p*-value
hsf-1 (*sy441*)	3	90 (17)	OP50	12.37 ± 0.34	control	
		90 (26)	OP50+Probio-M9	11.85 ± 0.46	-4.20%	0.7007

Total N, the number of total animals; censor N, the number of censored animals; Lifespan extension, the percentage of lifespan extension in different *C. elegans* mutants on OP50+Probio-M9 compared to those on OP50; *p* values, OP50+Probio-M9 versus OP50 by log rank test.

Supplementary Table 6. Lifespan analysis related to Figure S2.

C. elegans strain	Repeats	Total N (censor N)	Strain	Mean ± SEM (days)	Lifespan Extension	*p*-value
raga-1 (*ok386*)	1	90 (6)	OP50	16.79 ± 0.80	control	
		90 (7)	OP50+Probio-M9	23.15 ± 1.91	37.88%	< 0.0001
raga-1 (*ok386*)	2	90 (7)	OP50	16.11 ± 0.98	control	
		90 (4)	OP50+Probio-M9	22.46 ± 1.60	39.42%	< 0.0001
raga-1 (*ok386*)	3	90 (14)	OP50	16.69 ± 0.92	control	
		90 (18)	OP50+Probio-M9	24.59 ± 1.90	47.33%	< 0.0001
rsks-1 (*ok1255*)	1	90 (10)	OP50	23.93 ± 0.83	control	
		90 (14)	OP50+Probio-M9	29.94 ± 1.54	25.11%	< 0.0001
rsks-1 (*ok1255*)	2	90 (12)	OP50	22.60 ± 0.82	control	
		90 (8)	OP50+Probio-M9	30.73 ± 1.55	35.97%	< 0.0001
rsks-1 (*ok1255*)	3	90 (12)	OP50	23.21 ± 0.93	control	
		90 (25)	OP50+Probio-M9	29.38 ± 1.43	26.58%	< 0.0001

Total N, the number of total animals; censor N, the number of censored animals; Lifespan extension, the percentage of lifespan extension in different *C. elegans* mutants on OP50+Probio-M9 compared to those on OP50; *p* values, OP50+Probio-M9 versus OP50 by log rank test.

Supplementary Table 7. Lifespan analysis related to Figure 6.

C. elegans strain	Repeats	Total N (censor N)	Strain	Mean ± SEM (days)	Lifespan Extension	*p*-value
atfs-1 (*gk3094*)	1	90 (17)	OP50	11.50 ± 0.73	control	
		90 (19)	OP50+Probio-M9	11.83 ± 1.05	2.87%	0.8163
atfs-1 (*gk3094*)	2	90 (24)	OP50	12.83 ± 1.21	control	
		90 (21)	OP50+Probio-M9	13.19 ± 1.00	2.81%	0.8887
atfs-1 (*gk3094*)	3	90 (33)	OP50	11.45 ± 0.51	control	
		90 (31)	OP50+Probio-M9	12.03 ± 0.76	5.07%	0.1458
isp-1 (*qm150*)	1	90 (11)	OP50	22.34 ± 2.84	control	
		90 (14)	OP50+Probio-M9	22.31 ± 2.04	-0.13%	0.6027
isp-1 (*qm150*)	2	90 (8)	OP50	19.55 ± 1.77	control	
		90 (16)	OP50+Probio-M9	21.81 ± 2.95	11.56%	0.3454
isp-1 (*qm150*)	3	90 (29)	OP50	20.39 ± 2.06	control	
		90 (26)	OP50+Probio-M9	22.42 ± 2.53	9.95%	0.1839
nuo-6 (*qm200*)	1	90 (16)	OP50	32.61 ± 1.58	control	
		90 (4)	OP50+Probio-M9	33.93 ± 1.49	4.05%	0.9762
nuo-6 (*qm200*)	2	90 (12)	OP50	28.63 ± 1.41	control	
		90 (20)	OP50+Probio-M9	28.65 ± 1.55	0.07%	0.7502
nuo-6 (*qm200*)	3	90 (18)	OP50	30.62 ± 1.50	control	
		90 (24)	OP50+Probio-M9	30.71 ± 1.49	0.29%	0.9908

Total N, the number of total animals; censor N, the number of censored animals; Lifespan extension, the percentage of lifespan extension in different *C. elegans* mutants on OP50+Probio-M9 compared to those on OP50; *p* values, OP50+Probio-M9 versus OP50 by log rank test.

Supplementary Table 8. The strains and reagents used in this study.

Resource	Source	Identifier
Bacterial		
Escherichia coli: Strain OP50	CGC	WormBase: OP50
Lacticaseibacillus rhamnosus GG	Guangdong microbial culture preservation center	LGG
Experimental Models: Strains		
C. elegans: Strain: wild isolate	CGC	WormBase: N2
C. elegans: Strain: *nsy-1* (*ag3*) II	CGC	WormBase: AU3
C. elegans: Strain: *sek-1* (*km4*) X	CGC	WormBase: KU4
C. elegans: Strain: *tir-1* (*tm3036*) III	CGC	WormBase: IG685
C. elegans: Strain: *tir-1* (*ok1052*) III	CGC	WormBase: RB1085
C. elegans: Strain: *atf-7* (*gk715*) III	CGC	WormBase: VC1518
C. elegans: Strain: *eat-2* (*ad1116*) II	CGC	WormBase: DA1116
C. elegans: Strain: *skn-1* (*mg570*) IV	CGC	WormBase: GR2245
C. elegans: Strain: *atfs-1* (*gk3094*) V	CGC	WormBase: VC3201
C. elegans: Strain: *nuo-6* (*qm200*) I	CGC	WormBase: MQ1333
C. elegans: Strain: *isp-1* (*qm150*) IV	CGC	WormBase: MQ887
C. elegans: Strain: *rsks-1* (*ok1255*) III	CGC	WormBase: RB1206
C. elegans: Strain: *pmk-1* (*km25*) IV	Ye Tian	WormBase: KU25
C. elegans: Strain: *raga-1* (*ok386*) II	Ye Tian	WormBase: VC222
C. elegans: Strain: *daf-2* (*e1370*) III	Ye Tian	WormBase: CB1370
C. elegans: Strain: *daf-16* (*mgDf50*) I	Ye Tian	WormBase: GR1307
C. elegans: Strain: zcIs13[*hsp-6p::gfp*] V	Ye Tian	WormBase: SJ4100
C. elegans: Strain: zcIs4[*hsp-4p::gfp*] V	Ye Tian	WormBase: SJ4005
C. elegans: Strain: *aak-2* (*ok524*) X	Xiaoyun Xu	WormBase: RB754
C. elegans: Strain: *hsf-1* (*sy441*) I	Xiaoyun Xu	WormBase: PS3551
C. elegans: Strain: *age-1* (*hx546*) II	Xiaoyun Xu	WormBase: TJ1052
C. elegans: Strain: *dvIs70*[*hsp-16.2p::gfp* + *rol-6* (*su1006*)]	Xiaoyun Xu	WormBase: CL2070
C. elegans: Strain: *ldIs7*[*skn-1b/c::gfp* + *rol-6* (*su1006*)]	Xiaoyun Xu	WormBase: LD1
C. elegans: Strain: dvIs19 [(pAF15) *gst-4p::gfp*::NLS] III	Xiaoyun Xu	WormBase: CL2166

Supplementary Table 8 (continue). The strains and reagents used in this study.

Resource	Source	Identifier
Chemicals		
M.R.S. BROTH	OXOID	Cat#CM1175
TRYPTONE	OXOID	Cat#LP0042
YEAST EXTRACT	OXOID	Cat#LP0021
Bacto Agar	BD	Cat#214010
Bacto Peptone	BD	Cat#211677
Cholesterol	Sigma	Cat#C8503
Dimethyl sulfoxide	Sigma	Cat#SHBK2750
Tunicamycin	Abcam	Cat#ab120296
One-Taq Mix	NEB	Cat#M0484S
Softwares		
GraphPad Prism 8.0	GraphPad Software	https://www.graphpad.com/ scientificsoftware/prism/
ImageJ	Wayne Rasband (NIH)	https://imagej.nih.gov/ij/
FLUOVIEW FV1200 Viewer	Olympus	https://www.olympus-lifescience.com/en/
R software version 3.6.1. MetaboAnalyst	MetaboAnalyst	https://www.metaboanalyst.ca/
