# Supplementary material for: Lacticaseibacillus rhamnosus Probio-M9 extends the lifespan of Caenorhabditis elegans
Source: Commun Biol. 2022 Oct 27;5:1139. doi: 10.1038/s42003-022-04031-2 (PMC9613993; doi:10.1038/s42003-022-04031-2)
Supplement: Supplementary file 3 — Description of Additional Supplementary Files [file 42003_2022_4031_MOESM3_ESM.pdf]

Description of Additional Supplementary Files

File name: Supplementary Data 1

Description: The details summarized differentially regulated metabolites between OP50 and OP50+Probio-M9 when the change in metabolite level simultaneously met the criteria of a fold change (FC) ≤ 0.5 or ≥ 2 and a variable importance in projection (VIP) score threshold ≥ 1.

File name: Supplementary Data 2

Description: The source data used to create the box plots in the figures.

File name: Supplementary Data 3

Description: The source data used to create the survival curves in the figures.

File name: Supplementary Data 4

Description: The source data used to create the differentially metabolites between OP50 and Probio-M9 in the figures.
