# Supplementary material for: Lacticaseibacillus rhamnosus Probio-M9 extends the lifespan of Caenorhabditis elegans
Source: Commun Biol. 2022 Oct 27;5:1139. doi: 10.1038/s42003-022-04031-2 (PMC9613993; doi:10.1038/s42003-022-04031-2)
Supplement: Supplementary file 8 — Reporting Summary [file 42003_2022_4031_MOESM8_ESM.pdf]

nature portfolio

Corresponding author(s): Tiansong Sun

Last updated by author(s): Sep 12, 2022

Reporting Summary

Nature Portfolio wishes to improve the reproducibility of the work that we publish. This form provides structure for consistency and transparency in reporting. For further information on Nature Portfolio policies, see our Editorial Policies and the Editorial Policy Checklist.

Statistics

For all statistical analyses, confirm that the following items are present in the figure legend, table legend, main text, or Methods section.

n/a	Confirmed	
☐	☒	The exact sample size (n) for each experimental group/condition, given as a discrete number and unit of measurement
☐	☒	A statement on whether measurements were taken from distinct samples or whether the same sample was measured repeatedly
☐	☒	The statistical test(s) used AND whether they are one- or two-sided<br>*Only common tests should be described solely by name; describe more complex techniques in the Methods section.*
☐	☒	A description of all covariates tested
☐	☒	A description of any assumptions or corrections, such as tests of normality and adjustment for multiple comparisons
☐	☒	A full description of the statistical parameters including central tendency (e.g. means) or other basic estimates (e.g. regression coefficient) AND variation (e.g. standard deviation) or associated estimates of uncertainty (e.g. confidence intervals)
☐	☒	For null hypothesis testing, the test statistic (e.g. F, t, r) with confidence intervals, effect sizes, degrees of freedom and P value noted<br>*Give P values as exact values whenever suitable.*
☒	☐	For Bayesian analysis, information on the choice of priors and Markov chain Monte Carlo settings
☒	☐	For hierarchical and complex designs, identification of the appropriate level for tests and full reporting of outcomes
☒	☐	Estimates of effect sizes (e.g. Cohen's d, Pearson's r), indicating how they were calculated

Our web collection on statistics for biologists contains articles on many of the points above.

Software and code

Policy information about availability of computer code

Data collection: Confocal Images were obtained with Olympus FV1200, Japan.

Data analysis: GraphPad prism 8.0 was used to analyze the lifespan curves, and Log rank (Mantel-Cox) method was used to analyze the significance of the difference. For statistical data, Student's t test, Chi-squared test, one-way ANOVA and two-way ANOVA analysis coupled with multiple comparisons were used for significance analysis. In all cases, p < 0.05 was considered significant. In the figures, the asterisk indicates the statistical significance of the Log rank test, Student's t test, Chi-squared test, one-way ANOVA and two-way ANOVA analysis as compared to control. For metabolomics data, the OPLS-DA model was applied using R package "MetaboAnalyst". Permutation test repeated 200 times were used to verify this model, and p < 0.05 indicated the available OPLS-DA model. The significance of each metabolite was measured by Student's t test and fold change, and one-tailed Student's t test or Fisher's exact test were used to analyze the statistical significance, p < 0.05 was considered to be statistically significant. Using the Benjamini-Hochberg method, p value was corrected for multiple testing by false-discovery rate (FDR). ImageJ was used for image analysis. Database for Annotation, Visualization and Integrated Discovery (DAVID, http://david.ncifcrf.gov/) was used for KEGG pathway analysis.

For manuscripts utilizing custom algorithms or software that are central to the research but not yet described in published literature, software must be made available to editors and reviewers. We strongly encourage code deposition in a community repository (e.g. GitHub). See the Nature Portfolio guidelines for submitting code & software for further information.

Data

Policy information about availability of data

All manuscripts must include a data availability statement. This statement should provide the following information, where applicable:

- Accession codes, unique identifiers, or web links for publicly available datasets
- A description of any restrictions on data availability
- For clinical datasets or third party data, please ensure that the statement adheres to our policy

Raw data for lifespan analysis and differentially expressed metabolites analysis are provided in Supplementary Table.

Field-specific reporting

Please select the one below that is the best fit for your research. If you are not sure, read the appropriate sections before making your selection.

☒ Life sciences ☐ Behavioural & social sciences ☐ Ecological, evolutionary & environmental sciences

For a reference copy of the document with all sections, see nature.com/documents/nr-reporting-summary-flat.pdf

Life sciences study design

All studies must disclose on these points even when the disclosure is negative.

Sample size	Preliminary experiments to determine effect size were carried out. Animal numbers were chosen based on effect size and animal number available considerations regarding animal use.
Data exclusions	Caenorhabditis elegans, which accidentally lost, extruded organs or displayed matricidal hatching, were censored and excluded from lifespan analysis.
Replication	All reported data was successfully reproduced in independent replicate experiments.
Randomization	Animals were assigned to groups randomly.
Blinding	Investigators were not blinded during data collection or analysis.

Reporting for specific materials, systems and methods

We require information from authors about some types of materials, experimental systems and methods used in many studies. Here, indicate whether each material, system or method listed is relevant to your study. If you are not sure if a list item applies to your research, read the appropriate section before selecting a response.

Materials & experimental systems

n/a	Involved in the study	
☒	☐	Antibodies
☒	☐	Eukaryotic cell lines
☒	☐	Palaeontology and archaeology
☐	☒	Animals and other organisms
☒	☐	Human research participants
☒	☐	Clinical data
☒	☐	Dual use research of concern

Methods

n/a	Involved in the study	
☒	☐	ChIP-seq
☒	☐	Flow cytometry
☒	☐	MRI-based neuroimaging

Animals and other organisms

Policy information about studies involving animals; ARRIVE guidelines recommended for reporting animal research

Laboratory animals	Caenorhabditis elegans
Wild animals	All information on Caenorhabditis elegans strains used in this study is detailed in Supplementary Table 8.
Field-collected samples	All information on Field-collected samples used in this study is detailed in the methods section.
Ethics oversight	The protocol used in this study is detailed in the methods section.

Note that full information on the approval of the study protocol must also be provided in the manuscript.
